# Naturally Occurring Peer Support through Social Media: The Experiences of Individuals with Severe Mental Illness Using YouTube

**DOI:** 10.1371/journal.pone.0110171

**Published:** 2014-10-15

**Authors:** John A. Naslund, Stuart W. Grande, Kelly A. Aschbrenner, Glyn Elwyn

**Affiliations:** 1 The Dartmouth Institute for Health Policy and Clinical Practice, Dartmouth College, Hanover, New Hampshire, United States of America; 2 The Dartmouth Center for Health Care Delivery Science, Dartmouth College, Hanover, New Hampshire, United States of America; 3 Department of Psychiatry, Geisel School of Medicine at Dartmouth, Hanover, New Hampshire, United States of America; Academic Medical Centre Amsterdam, Netherlands

## Abstract

Increasingly, people with diverse health conditions turn to social media to share their illness experiences or seek advice from others with similar health concerns. This unstructured medium may represent a platform on which individuals with severe mental illness naturally provide and receive peer support. Peer support includes a system of mutual giving and receiving where individuals with severe mental illness can offer hope, companionship, and encouragement to others facing similar challenges. In this study we explore the phenomenon of individuals with severe mental illness uploading videos to YouTube, and posting and responding to comments as a form of naturally occurring peer support. We also consider the potential risks and benefits of self-disclosure and interacting with others on YouTube. To address these questions, we used qualitative inquiry informed by emerging techniques in online ethnography. We analyzed n = 3,044 comments posted to 19 videos uploaded by individuals who self-identified as having schizophrenia, schizoaffective disorder, or bipolar disorder. We found peer support across four themes: minimizing a sense of isolation and providing hope; finding support through peer exchange and reciprocity; sharing strategies for coping with day-to-day challenges of severe mental illness; and learning from shared experiences of medication use and seeking mental health care. These broad themes are consistent with accepted notions of peer support in severe mental illness as a voluntary process aimed at inclusion and mutual advancement through shared experience and developing a sense of community. Our data suggest that the lack of anonymity and associated risks of being identified as an individual with severe mental illness on YouTube seem to be overlooked by those who posted comments or uploaded videos. Whether or not this platform can provide benefits for a wider community of individuals with severe mental illness remains uncertain.

## Introduction

How might the phenomenon of individuals with severe mental illness (SMI) such as schizophrenia, schizoaffective disorder, or bipolar disorder, who share their illness experiences through video narratives uploaded onto platforms like YouTube constitute the development of a naturally emerging peer support system? Or does this activity represent a risky process of self-exposure, and susceptibility to negative comments, unsolicited content, or exposure to harmful influences of others?

YouTube is a video-sharing website created in 2005 that is currently the third most popular social media website worldwide after Facebook and Google+ [1]. YouTube has videos uploaded in 61 different languages, and reaches more adults ages 18–34 in the United States than any cable network [2]. YouTube is free to use, and any individual with access to the Internet can upload videos, post comments, or share videos with others. While the potential harms and benefits of connecting with others to share health information through social media have been discussed extensively [3]–[5], diverse patient groups with sensitive health conditions are increasingly turning to social media websites like YouTube to share their illness experiences or seek advice from others with similar conditions [4], [6]. As people with diverse health conditions seek greater online connectivity, it is not clear whether YouTube may represent a platform by which individuals with SMI can provide and receive peer support or whether unforeseen risks might prevent such support.

Peer support in SMI includes a system of mutual giving and receiving where individuals who have faced and endured the adversity of mental illness can offer hope, companionship, and encouragement to others facing similar conditions [7], [8]. Peer support has been recognized as a promising strategy for mental health recovery and for overcoming limitations of illness [7]–[10]. However, much of the literature has focused on formal peer support programs delivered within community settings such as mental health centers or psychosocial clubhouses, as well as through Internet support groups, or provided in conjunction with existing evidence-based practices [8], [10]–[12]. Less attention has been given to informal peer-to-peer relationships spanning an individual’s community, surroundings, personal life, and social network [13]. Also, with the increasing popularity of social media and its growing importance as a means to connect and interact with others, there is a need to consider whether informal peer support naturally occurs outside of professional services and across a popular social media website such as YouTube.

Evidence suggests individuals with SMI are more likely to share personal views through blogging, build friendships on social media [14], and use the Internet for accessing health information [15] than people without mental illness. This could be in part because social media is perceived as a non-threatening medium that provides opportunities to identify and connect with similar individuals within the safety of one’s own home. Yet, uncertainty exists on how social media might be used for online peer support among individuals with SMI [16]. Our search of relevant literature did not identify any studies that characterize or explore types of social interactions or discussion content between peers with SMI on social media websites. Therefore an exploration of these interactions and how they might represent naturally occurring peer support among individuals with SMI is needed.

In the context of YouTube, groups of individuals with diverse health conditions, including multiple sclerosis [17], inflammatory bowel disease [18], or cancer [19], use this platform as a tool for searching for health information [20] and as a forum for sharing personal illness stories or receiving feedback and social support from others. For example, analyses of videos on YouTube generated by people with multiple sclerosis and corresponding comments demonstrate that many individuals frequently disclose personal health information in this online environment [21], and upload videos to provide treatment advice to others and share personal experiences seeking and obtaining medical care [17]. With evidence documenting the widespread use of YouTube among numerous patient groups, in the current study we aim to explore comments posted to YouTube by individuals who self-identify as having a SMI, taking the perspective that naturally occurring peer support through social media may be beneficial. Our objective is to observe how individuals with SMI interact on YouTube with their peers, knowing the risks of disclosure, and whether this might serve as a way to manage their own recovery and provide support to others.

First, to investigate this phenomenon of growing social media use among individuals with SMI, we provide a brief conceptual background to frame peer support and social media within the context of SMI. We then outline our process of exploring the comments associated with uploaded YouTube videos for expressions of peer support. Our analysis details how individuals with SMI use YouTube to interact with each other, and our discussion reflects the value of this popular social media website as a naturally occurring peer support platform.

### Peer Support and Severe Mental Illness

Peer support in psychiatry has its roots in the mental health consumer movement of the 1970s, where individuals living with SMI campaigned for basic human rights, mental health care reform, and the right to make their own health care choices [8], [22]. These advocacy efforts were in large part a protest against discrimination and injustices, including authoritarian psychiatric practices, compulsory hospitalizations, and coercion to undergo unproven treatments [23]. This activism led to collective action, and to the development of mutual support and self-help activities [8]. Central tenets of peer support in mental health include reciprocity, mutual respect, and shared responsibility [7]. For this highly stigmatized group [24], peer support offers optimism and hope for a better life and acceptance as well as opportunities to relate to one another through common experiences and shared struggles [25]. Relationships between peers are based upon commitment to positive personal growth and wider contribution to community building toward achieving mental health recovery [7].

Recent decades have seen the emergence and demonstrated effectiveness of formal peer provided services [11] including programs targeting mental health recovery [26], medical illness self-management [27], [28], and smoking cessation [29]. These programs are often guided by conventional therapeutic boundaries [25], and are delivered in an organized manner involving formal providers or in conjunction with prescribed medical and mental health services [13]. Structured programs fall in between natural and therapeutic relationships, and differ from informal peer support [25].

Informal peer support relationships are considered the foundation of peer support in mental health [30], and occur naturally between individuals who come together through shared experience. Informal peer support creates a sense of belonging and friendship [30], and allows individuals with SMI to feel greater self-worth and self-efficacy by supporting and impacting the life of another peer [25]. However, peers who provide support can also become overwhelmed or frustrated consistent with caregiver distress, or face interpersonal conflicts due to the burden of managing their own symptoms and mental health concerns [25]. As with any informal interactions, recommendations or advice provided by peers are based upon personal experiences, which at times might contradict professional recommendations or the content of structured programs. Despite these concerns, the benefits of informal peer support in SMI are widely accepted [7], [8].

### Social Media and Peer Support

Social media websites have afforded new opportunities for individuals to connect with others, provide and receive support, and share their illness experiences [4]. There are disease specific support groups on Facebook [31], [32], personal video logs posted by patients onto YouTube [21], and entire interactive forums dedicated to sharing illness experiences such as PatientsLikeMe [33]. Despite this widely reported use among the general population, use of popular social media among individuals with SMI has received less attention.

Evidence suggests that individuals with SMI create online relationships at the same rate [34] or higher when compared to individuals without a mental illness [14]. A case study examined the therapeutic benefit of using Facebook to reach an isolated patient with psychiatric comorbidities and foster social relationships [35]. Ethical considerations related to suicidal postings to Facebook among individuals with SMI, including privacy concerns and threats to the therapeutic alliance, have also been explored [36]. However, no prior studies have highlighted any specific risks of social media use among individuals with SMI that extend beyond what would be observed in general patient populations.

Social media may be well suited for peer support among individuals with SMI. These websites facilitate natural community building, where control of entry and exit are independent of social interaction, which allows users to avoid the anxiety and fears associated with face-to-face interactions [34]. With relative anonymity, individuals with SMI can find peers and discuss aspects of living with mental illness in a controlled and personal environment by using the Internet from home [37]. Also, individuals who are stigmatized because of their illness are often highly motivated to seek others with the same condition [38], something that social media can enable.

To date, however, the benefits and understanding the why and motivation of online peer support in SMI have not been established. We know that disclosure of personal information through social media has been linked to individual behavioral control [39] and psychological well-being [40]. One study [16] of unmediated online peer support for individuals with psychiatric disabilities, comparing a listserv and a bulletin board, replicated the spontaneous nature of Internet forums. The authors’ found many participants reported both positive experiences and greater distress, suggesting that online peer support may not deliver all intended benefits [16]. The uncertainty of these findings underscores the need to more fully explore the interactions between individuals with SMI on a popular social media website like YouTube to gain insight about informal peer support relationships and to observe whether harms or benefits emerge within an unmonitored and public online platform.

## Methods

Recognizing the contribution of social media on health and help seeking behaviors among individuals with SMI we planned an investigation of YouTube. Specifically, we explore uploaded YouTube videos and associated comments for expressions of peer support. We conducted a qualitative analysis of comments detailing how individuals with SMI use YouTube as a platform indicative of naturally occurring peer support.

### Online Ethnography for Exploring YouTube

Ethnographic methods in health research focus on expanding our understanding of illness experience and social practices in the context of culture within particular groups [41], [42]. Our approach was informed by an online ethnography framework [42], [43], where we observed online interactions on YouTube without interfering in natural conversations. Observer interference is typically a limitation of face-to-face observational methods [43]. In contrast to offline ethnographic methods consisting of extensive fieldwork [41], YouTube served as the research field where interactions reflective of peer support among individuals who self-identify as having a SMI were explored.

### Study Sample and Data Collection

In June 2013 we searched YouTube for publicly available videos that had been extensively viewed using the following search terms: “*mental illness*”, “*schizophrenia*”, “*schizoaffective disorder*” or “*bipolar disorder*”. To systematically identify a sample of relevant videos, we screened the first 100 videos for each search term. Relevant videos were defined as: posted by an individual, had no advertising, were in the first person, and included our search terms. Also, for each relevant video identified, YouTube generates a page list of 20 recommended videos that describe a similar topic based on our search history and the prior videos we selected and viewed. We reviewed the recommended video lists to identify additional relevant videos.

After viewing relevant videos, we further selected a smaller set of videos based on the following criteria: had a minimum of 5,000 views because videos with more views have more comments [18]; uploaded by an individual who self-identified in the video as having a SMI, either schizophrenia, schizoaffective disorder, or bipolar disorder (these diagnoses are associated with greater disability and stigma [24]); no evidence of involvement of public or private institutions or agencies in the creation of the video; video content detailed experiences living with SMI; and in English. We exported the associated comments for each video, posted by either commenters who self-identified as having a SMI, or video authors (i.e., individuals who uploaded the videos), to a pdf document for thematic analysis.

### Ethics

There has been considerable discussion surrounding the ethical concerns of analyzing data retrieved from social media [44], [45], including examples where the privacy of users has been at risk [46]. To minimize these concerns, we searched for and included only videos that were publicly available on YouTube. To further protect the identity of commenters and video authors we removed all usernames and did not include video titles or specific demographic characteristics in the results reported here. We also received institutional review board exemption from the Dartmouth College Committee for the Protection of Human Subjects to conduct this study.

### Qualitative Analysis

The comments associated with the smaller set of identified videos were then exported as a pdf document into ATLAS.ti software Version 7.0 for thematic analysis. We applied an open coding approach, and developed codes based on the data as they naturally occurred [47]. Informed by grounded theory, we determined concepts and themes related to expressions of peer support based on our analysis where those that were repeated across multiple comments were considered meaningful [48]. This approach has been applied in similarly designed research using online data [49]. We independently coded the comments, and then compared code lists. After combining and reviewing the code lists, we grouped similar codes into categories and resolved code disagreements by consensus. We then condensed the categories into broad themes based on ideas of how peer support exists. Given the exploratory nature of this project, we modeled Patton’s (1990) approach for summarizing qualitative results by presenting our data as they appeared on YouTube so that readers may also draw their own interpretations regarding the potential value of YouTube as a platform for informal peer support among individuals with SMI [50].

## Results

Our search of YouTube yielded a combined total of approximately 746,200 videos. After screening 400 videos (100 per search term), we followed the recommended other video lists (YouTube) and evaluated 1,100 potential videos. We narrowed this set down to 63 by selecting only videos posted by an individual, without advertising, in the first person. We then applied our strict inclusion criteria and selected 19 videos and their associated 3,044 comments for analysis. The 44 excluded videos did not meet our criteria either because they did not have enough views, did not describe experiences of living with SMI, or appeared to be uploaded by private companies, documentary filmmakers, or academic institutions.

The 19 videos were uploaded by 19 different individuals of which 8 self-identified as being diagnosed with bipolar disorder, 7 with schizoaffective disorder, and 4 with schizophrenia. Common topics included managing illness symptoms, challenges and benefits of seeking treatment, coping strategies for day-to-day concerns, efforts to reach out and help others with similar conditions, and personal stories about life goals, interests and future ambitions. The videos had an average of 19,786 views (range of 5,433 to 88,654) and had been on YouTube between 87 and 1,798 days. Fifteen videos appeared to be posted by young adults (18–35 years) and four by middle-aged adults (36–55 years). We analyzed 3,044 comments posted to the videos, ranging from 27 to 592 for each video, of which 91% were posted by commenters and 9% were posted in response to commenters by video authors. On YouTube, users can rate comments as ‘inappropriate’, and when a comment receives sufficient ‘inappropriate’ ratings it is flagged. About 5% (n = 160) of the comments we analyzed were flagged for content viewed as inappropriate such as derogatory or discriminatory statements, advertisements, or spam.

We determined four themes representing key aspects of peer support (see Table 1). The themes are discussed below and examples of unedited comments are cited to provide context.

**Table 1:** Themes representing peer support.

*Themes reflecting expressions of naturally occurring peer support on YouTube*
1. Minimizing a sense of isolation and providing hope
2. Finding support through peer exchange and reciprocity
3. Coping with the day-to-day challenges of severe mental illness
4. Learning from shared experiences of medication use and seeking mental health care

### Minimizing a sense of isolation and providing hope

Efforts to reduce social isolation and achieve a sense of community were expressed in comments posted across all 19 videos. Comments showed appreciation for individuals who shared personal histories and lived experiences. Many commenters acknowledged that having a mental illness is a “lonely road” and were thankful for the videos, conveying reassurance to know that they are not alone with their illness. Individuals who said they felt that they could not talk to anyone about their illness because of fear, embarrassment or simply not having anyone close to them in their lives were particularly positive:

Thank you soo much for this video it has made me feel a little less alone … i myself have bipolar 2 and have been off meds for about 6 months now and am also feeling the day to day fight i have in my head just to live in the real world …

*Comment on Video #5: video uploaded by female with bipolar disorder*

I can relate to almost everything you say … I’m too afraid to talk to people to have someone to relate to and discuss it with. So this video helps me in that I get to listen to your story and what you’re going through and feel like I’m not alone …

*Comment on Video #18: video uploaded by male with schizophrenia*

Peer support also allows individuals with SMI to relate to one another based on shared experiences and perceived membership in a wider supportive network. For some, this sense of belonging came from a sense of normalcy after watching the personal stories shared by others:

I’m very happy i found you and your videos … Its so good to find people that make me think I’m more “normal” than i think. Thank you and hope alls good!

*Comment on Video #17: video uploaded by female with schizophrenia*

For some, viewing the videos was a deeply emotional experience, generated by a sense of relief of finding someone to relate to:

Literally, tears were streaming down my face as I watched this. Thank you so much … your descriptions were so spot on … it felt as if a female version of me from the future made this video … for no other reason but to give helpful advice.

*Comment on Video #17: video uploaded by female with schizophrenia*

The YouTube videos also served as a source of inspiration, prompting individuals to do the same, and share their own illness stories. The importance of the Internet as a means for reaching others facing similar challenges was frequently cited:

It’s okay, you’re not alone, there are many of our kind and we are lost without each other, but now thanks to the internet we can reach out to each other so we can discuss what we hear openly and honestly with no fear of being judged …

*Comment on Video #3: video uploaded by female with schizoaffective disorder*

I’m thinking about… making videos about my illness so I can connect with you and others here on youtube …

*Comment on Video #16: video uploaded by female with schizophrenia*

Comments also reacted to the idea of sensing hope, expressed as optimism for overcoming challenges and moving forward with their lives:

I can completely relate to your experience. I was diagnosed with Schizophrenia about two years ago and it has been hell for me. Your video was very inspiring to me and it gave me hope that things can get better.

*Comment on Video #16: video uploaded by female with schizophrenia*

### Finding support through peer exchange and reciprocity

Comments also contained offers of comfort and encouragement. One commenter reassured “hang in there, things can and will get better … and thank you for sharing your story with us” (*Comment on Video #6: video uploaded by female with schizoaffective disorder*). Another suggested “Together us bipolar people will survive. It’s all about support. We will find a cure” (*Comment on Video #1: video uploaded by female with bipolar disorder*).

Insightful dialogues occurred between commenters. In the YouTube environment participants have the opportunity to share their opinions, mirroring the democratic process of giving and receiving that defines informal peer support interactions. For example, discussions emerged about various topics related to SMI, as well as personal anecdotes and common interests like favorite music or art, illustrating the development of forms of social relationships.

Hey man I can really relate to what you say about being hospitalized and meeting a girl there, and I have schizo affective disorder, and its rough … The Beatles are my favorite band too man.

*Comment on Video #18: video uploaded by male with schizophrenia*

Some commenters reached out to video authors to seek additional support for personal concerns outside the public YouTube network. For example, a commenter asks whether the person who uploaded the video would be willing to talk using Skype:

Would you be opposed to Skyping with someone who wasn’t sure about the symptoms they’re starting to have? I’ve just started hearing voices. I don’t have anyone to talk to about this.

*Comment on Video #19: video uploaded by male with schizophrenia*

Commenters rapidly defended individuals who uploaded a video when someone posted a negative comment. This illustrates reciprocity. These negative comments about the video content or the individual elicited strong responses from other commenters overwhelmingly in defense of the video author. One negative commenter stated “bipolar disorder is not real” and that psychiatrists are “evil”, claiming that people with mental illness are all “drug addicts”:

You people … make these vids while your high as a kite to ‘prove’ you have bipolar. Just shows how fake this fucking disease is …

*Comment on Video #3: video uploaded by female with bipolar disorder*

Many comments responded immediately and in strong terms:

If u actually suffered from bipolar disorder you wouldn’t be saying such evil crap it is absolute hell it affects everything and by the way … i am bipolar … so u can shove that drug addicted crap up your ass … keep your hateful insensitive comments to yourself you don’t know shit

*Comment in response to negative comment on Video #3: video uploaded by female with bipolar disorder*

Interactions such as these demonstrate the solidarity created and efforts made to protect the network of those willing to openly share their experience of living with SMI.

### Coping with the day-to-day challenges of severe mental illness

YouTube appears to overcome the perceived inability of some people with SMI to learn how to cope, by providing users with the opportunity to share thoughts, struggles, hopes and fears in a receptive network. Commenters admitted to the struggles of living with mental illness and said that posting comments to YouTube may, in itself, serve as a coping mechanism. For some viewers, expressing personal challenges to peers that may be listening appears to be a way to search for validation and elicit supportive responses from others. One commenter wrote:

I hate being bipolar, it’s been the biggest burden by far and it’s not the first time I’ll say I’m sick of it. I hate having no control over myself … Stuck in your own head is a good way to put it… I dread knowing it’s never going away.

*Comment on Video #7: video uploaded by male with bipolar disorder*

Others were more graphic in their depiction of living with SMI, and spared no details in illustrating the reality of their condition:

Sometimes i get anxious as fuck and angry as shit for no reason. i chain smoke two packs worth of cigarettes a day that i handroll myself. The medications made me a fat bastard at 310 pounds and i cant keep up my grooming worth a shit. i still manage to hold down a job and i still live in a group home. there thats my fucking life story. your welcome everyone:)!

*Comment on Video #2: video uploaded by female with schizoaffective disorder*

Extensive discussions emerged on how to cope with SMI. As individuals described their day-to-day challenges many reflected on the strategies that have helped them to cope and achieve a sense of “normalcy” in their everyday lives. Some commenters recommended coping strategies to each other, or asked for advice for dealing with their disorder. One viewer described how counseling serves as an important coping mechanism, “I go to counseling and that helps more than talking to anyone around me in my life because… the counselors at least have been around more bipolar people” (*Comment on Video #5: video uploaded by female with bipolar disorder*).

Individuals also shared their personal experiences of how to cope, e.g. practicing music:

Hey man, i wanted to let you know that you have a lot of courage for making this video. You really should find a way to cope with both the manic and depressive episodes. May i suggest an instrument? I picked up the guitar a few years ago to help me cope with my excessive energy during manic states, and it has done wonders, it has helped me more than any drug or any therapy.

*Comment on Video #3: video uploaded by male with bipolar disorder*

### Learning from shared experiences of medication use and seeking mental health care

Commenters helped others better understand what it is like to receive mental health care and even with overcoming fears by sharing lessons learned of using medications and seeking mental health care before or after receiving a mental illness diagnosis. Comments contained detailed accounts of taking medications and filling out prescriptions, and of being hospitalized, visiting mental health providers and medical professionals, and facing difficult diagnoses. There were positive views such as praise for psychiatrists and emphasis on the importance of having a good relationship with mental health providers. One commenter emphasized the importance of taking medications:

The biggest mistake we can make is to just stop taking your meds. It happens to the best of us. You “think” I’m feeling good I don’t need these meds. Not true! I know now that I NEED my meds to be well. It sucks, but it is what it is.

*Comment on Video #5: video uploaded by female with bipolar disorder*

There were also many examples of frustration with receiving mental health care, describing the negative side effects of taking medications, fear of taking medications, experiences of being hospitalized, and not getting a clear diagnosis from different mental health providers. This individual explains the fear of taking medications:

This is the best video so far on this topic. Nicely done! I have been going through this battle since I was 11 years old, I am now 23. I currently am not on medication. I’m scared to get back on them. The side effects highten my symptoms …

*Comment on Video #14: video uploaded by male with schizoaffective disorder*

The following commenter describes the negative consequences of a wrong diagnosis until finally finding the *right* doctor:

You have only been in the hospital one time? Girl, you really are fortunate. I have been in and out of hospital(s) for the last 12 years. Several attempted suicides due to the fact that it took the “right” doctor to come into my path and finally diagnose me with Bipolar Disorder rather than your standard “Major Clinical Depression”

*Comment posted to Video #8: video uploaded by female with bipolar disorder*

Comments highlighted individuals’ fear of visiting doctors because of the diagnosis that they might get or risk of being institutionalized. Individuals used YouTube to seek advice:

i have conversations with my self and sometimes only notice what i have done when i stop talking… i also have violent urges out of the blue but i can control them at the moment. i know these are signs of schizerphrnia but im seeing my psychiatrist in 5 days an wondering if i should tell him … i dont want to be put in a mental ward will he put me in there if i tell him these things ???? please respond worried

*Comment on Video #1: video uploaded by female with schizoaffective disorder*

The video author responded:

he will DEFINITELY not be able to lock you up in psychiatric unit if you tell him what you’ve been experiencing. The only way they could send you to the hospital (this is what I’ve experienced, anyways) is if you tell them that you’ve been hearing voices telling you to harm yourself or others, or if you have been having suicidal thoughts. … you should really tell your psychiatrist about this, though!!!

*Response to Comment on Video #1: video uploaded by female with schizoaffective disorder.*

Individuals with SMI appear to use YouTube to seek and share advice about medical related questions and concerns, which might or might not suggest they have been unable to find answers elsewhere. It is also possible that these individuals are looking to validate health information found elsewhere, such as from a recent medical visit or content from health-related websites, which led to their discovering personal accounts from others on YouTube. Whether these individuals feel most comfortable consulting with peers through YouTube, or perhaps use other social media platforms for the same purpose, remains unclear. Regardless, a rich dialogue surrounding shared experiences using medications and seeking mental health care emerged, illustrating another practical reason for engaging in these online peer-to-peer interactions through YouTube.

## Discussion

The reciprocal exchange between YouTube commenters in response to content uploaded by individuals who self-identify as having a SMI including schizophrenia, schizoaffective disorder, or bipolar disorder, represents a dynamic, responsive, yet informal, system of peer support. Analysis of the data provided four themes consistent with the central role of peer support in SMI: minimizing a sense of isolation and providing hope; finding support through peer exchange and reciprocity; sharing strategies for coping with the day-to-day challenges of severe mental illness; and learning from shared experiences of medication use and seeking mental health care. We see peer support on YouTube as voluntary–an emergent phenomenon where users are unhindered and have individual autonomy to choose their level of disclosure and engagement, from viewing to commenting to uploading personal videos.

Personal health information was frequently shared and rewarded by positive, encouraging, and insightful viewer responses. These interactions created, what appeared to be, a sense of belonging and means for coping with challenges of SMI. We observed that YouTube served as an environment where individuals with SMI could normalize one’s illness and assert their voice and identity by validating shared experiences with peers. That individuals were able to do this suggests the degree of safety they felt in contributing their videos and comments. This “peer exchange” was reinforced by strongly supportive, and at times combative, responses of participants in reaction to negative comments or “outsider” attempts to criticize uploaded videos.

The observation of real world data without researcher interference was a methodological strength because it allowed us to document and analyze naturally occurring interactions. There were also limitations with our methods because we could not obtain data on individual motivation or intention to upload videos, post comments, or publicly disclose personal health information. This limitation with our data also restricted our ability for more in depth interpretation and analysis of the content posted to YouTube because of increased risk of bias. A previous study of YouTube found viewers were more comfortable commenting on lay videos, citing that the value of interacting with someone with firsthand experience meant it was someone who may be more likely to respond to questions or give advice [18]. Further, we were unable to assess the impact of the videos or comments on the actual individuals with SMI, such as whether the interactions through YouTube offer long-term benefit and genuine support, or whether it was a distraction. There was also no way for us to test out possible harms of using this medium as these were not mentioned by commenters, though we contend that some harms likely do exist. Because of the qualitative nature of this study, we are unable to provide detailed metrics for the included videos and comments, or analysis for patterns within the data, such as between type of comments and number of views, or between positive and negative comments. Lastly, this was an exploratory study and we are unable to generalize our findings to a wider population of individuals with SMI or reach definite conclusions regarding the benefits of naturally occurring peer support on YouTube.

Peer-to-peer interactions and open sharing of personal experiences living with SMI through YouTube exemplify how social media and the Internet are reshaping the way this marginalized group engages their environment and seeks and shares advice related to mental health care. As highlighted in prior research, by sharing illness experiences with others online and through social media, there is potential for improved health and psychosocial outcomes, including benefits such as learning from others, feeling supported, and forming relationships [51]. For example, individuals with depression reported using online support groups to elicit support and empathy from others, express themselves, and serve as a form of community building [52]. Among individuals living with HIV/AIDS, perceived benefits of online peer-to-peer interactions range from seeking informational and emotional support [53], [54], to achieving greater empowerment through gathering information, finding positive meaning, receiving social support, and helping others [55].

Then again, other studies found no clear health benefit of peer-to-peer interactions in social media [56]. The presence of promotional material and advertisements may have limited these prior findings [31] by emphasizing awareness and fundraising efforts rather than how individuals share illness experiences and reciprocate peer support [32]. Our findings differed from these prior studies because only a small number of comments from our YouTube sample were flagged as inappropriate or as spam while comments promoting illness awareness or fundraising were not widely visible. This may have occurred because we selected only videos uploaded by individuals who self-identified as having schizophrenia, schizoaffective disorder, or bipolar disorder, as opposed to videos uploaded by foundations, companies, health care organizations or research institutions. While any subsequent exploration using our inclusion criteria would likely find different content, we believe the nature of peer support would be consistent.

As the benefits of social media for individuals with SMI remain unclear, how might sharing experiences and interacting with peers through YouTube specifically support mental health recovery? Listening to the experiences of other individuals with SMI offers value because authentic accounts are easier to understand, can make treatment information more relevant by putting it into a personal context, can help make sense of one’s situation, and can help with overcoming fears and feeling more confident in coping with symptoms [51], [57]. Also, social media facilitate connections between individuals facing similar health challenges, which creates a sense of community and belonging, helps people feel more “normal”, fosters personal empowerment, and provides socially isolated individuals with opportunities to feel connected and less alone [51], [58]. Though compelling, future research is needed to better understand whether informal peers openly sharing illness experiences on social media can support mental health recovery in individuals with SMI. In this context, it is “recovery in mental illness” where the emphasis for individuals is on learning to live with their illness while feeling empowered to pursue personal life goals, as opposed to “recovery from mental illness” where amelioration of symptoms and improved daily functioning are the primary focus [59].

Our findings reflect preliminary data that characterize naturally occurring interactions among individuals with SMI through social media and we acknowledge this new medium of communication poses potential risks. For instance, uncertainty remains about what level of information is necessary to determine what an online peer is saying is trustworthy and applicable [60]. On YouTube, perhaps number of video views determines perceived trustworthiness of the content. Alternatively, physical appearance of the person who uploaded the video may contribute to perceived trustworthiness, where an attractive or friendly looking individual may be considered more reliable. Given the largely unregulated nature of YouTube, there is also risk that all sources may be considered equivalent regardless of their trustworthiness [51]. Future research should seek to determine the trustworthiness of the videos as well as the authenticity of the video authors’ and commenters’ self-identified SMI diagnoses.

Another concern might be that dramatic videos receive more attention through increased views and visibility while routine or unremarkable experiences may receive comparatively little notice [51]. In examining our included videos, we did not observe major differences in tone or content based on the number of views or comments, suggesting such concerns were not present. Yet, consistent with other online networks, clear challenges of balance through YouTube are expected [61]. Additional risks from accessing content on YouTube may include increased anxiety or confusion about one’s own symptoms or condition, feeling inferior if others are doing better, unrealistic expectations regarding treatment, and exposure to misleading information. However, these varied risks are not unique to YouTube and could arise in cases where the credentials of the individuals providing advice are not known. As suggested by our data, the potential benefits of interacting with peers online outweigh any potential downsides [51], and the use of YouTube within this vulnerable population group represents an emerging dimension of social life worthy of investigation.

## Conclusion

At the time of this study, among the individuals who self-identified as having a SMI and who uploaded videos or posted comments to YouTube, we observed a sense of reward emerging from their interactions, mutual learning, and offered peer support. Of importance is our finding that peer support is happening naturally among individuals with highly stigmatized psychiatric illnesses within an unmonitored and public online platform. The lack of anonymity and associated risks of being identified as an individual with SMI seemed to be overlooked by commenters and video authors. Whether or not this platform can provide benefits for a wider group of individuals with SMI remains uncertain.

## References

[1] Kosner AW (2013) Watch out Facebook, with Google+ at #2 and YouTube at #3, Google, Inc. could catch up. Forbes. Available: http://www.forbes.com/sites/anthonykosner/2013/01/26/watch-out-facebook-with-google-at-2-and-youtube-at-3-google-inc-could-catch-up/.

[2] YouTube (2014) YouTube Statistics. Available: http://www.youtube.com/yt/press/statistics.html.

[3] Chou W-YS, Hunt YM, Beckjord EB, Moser RP, Hesse BW (2009) Social media use in the United States: implications for health communication. Journal of Medical Internet Research 11: e48. 19945947 10.2196/jmir.1249 PMC2802563

[4] Vance K, Howe W, Dellavalle RP (2009) Social internet sites as a source of public health information. Dermatologic Clinics 27: 133–136. 19254656 10.1016/j.det.2008.11.010

[5] Moorhead SA, Hazlett DE, Harrison L, Carroll JK, Irwin A, et al (2013) A new dimension of health care: systematic review of the uses, benefits, and limitations of social media for health communication. Journal of Medical Internet Research 15: e85. 23615206 10.2196/jmir.1933 PMC3636326

[6] Fox S, Jones S (2009) The social life of health information. Washington, DC: Pew Internet & American Life Project: 2009–2012.

[7] Mead S, Hilton D, Curtis L (2001) Peer support: a theoretical perspective. Psychiatric Rehabilitation Journal 25: 134–141. 11769979 10.1037/h0095032

[8] Davidson L, Chinman M, Sells D, Rowe M (2006) Peer support among adults with serious mental illness: a report from the field. Schizophrenia Bulletin 32: 443–450. 16461576 10.1093/schbul/sbj043 PMC2632237

[9] Anthony WA (1993) Recovery from mental illness: the guiding vision of the mental health service system in the 1990s. Psychiatric Rehabilitation Journal 16: 11–23.

[10] Solomon P (2004) Peer support/peer provided services underlying processes, benefits, and critical ingredients. Psychiatric Rehabilitation Journal 27: 392–401. 15222150 10.2975/27.2004.392.401

[11] Chinman M, George P, Dougherty RH, Daniels AS, Ghose SS, et al (2014) Peer support services for individuals with serious mental illnesses: assessing the evidence. Psychiatric Services 65: 429–441. 24549400 10.1176/appi.ps.201300244

[12] Coniglio FD, Hancock N, Ellis LA (2012) Peer support within Clubhouse: A grounded theory study. Community Mental Health Journal 48: 153–160. 20972830 10.1007/s10597-010-9358-5

[13] Davidson L, Chinman M, Kloos B, Weingarten R, Stayner D, et al (1999) Peer support among individuals with severe mental illness: a review of the evidence. Clinical Psychology: Science and Practice 6: 165–187.

[14] Gowen K, Deschaine M, Gruttadara D, Markey D (2012) Young adults with mental health conditions and social networking websites: seeking tools to build community. Psychiatric Rehabilitation Journal 35: 245–250. 22246123 10.2975/35.3.2012.245.250

[15] Berger M, Wagner TH, Baker LC (2005) Internet use and stigmatized illness. Social Science & Medicine 61: 1821–1827. 16029778 10.1016/j.socscimed.2005.03.025

[16] Kaplan K, Salzer MS, Solomon P, Brusilovskiy E, Cousounis P (2011) Internet peer support for individuals with psychiatric disabilities: a randomized controlled trial. Social Science & Medicine 72: 54–62. 21112682 10.1016/j.socscimed.2010.09.037

[17] Mazanderani F, O’Neill B, Powell J (2013) “People power” or “pester power”? YouTube as a forum for the generation of evidence and patient advocacy. Patient Education and Counseling 93: 420–425. 23830239 10.1016/j.pec.2013.06.006 PMC3863946

[18] Frohlich DO, Zmyslinski-Seelig A (2012) The presence of social support messages on YouTube videos about inflammatory bowel disease and ostomies. Health Communication 27: 421–428. 21962112 10.1080/10410236.2011.606524

[19] Chou W-YS, Hunt Y, Folkers A, Augustson E (2011) Cancer survivorship in the age of youtube and social media: a narrative analysis. Journal of Medical Internet Research 13: e7. 21247864 10.2196/jmir.1569 PMC3221357

[20] Freeman B, Chapman S (2007) Is “YouTube” telling or selling you something? tobacco content on the YouTube video-sharing website. Tobacco Control 16: 207–210. 17565142 10.1136/tc.2007.020024 PMC2598506

[21] Fernandez-Luque L, Elahi N, Grajales III FJ (2009) An analysis of personal medical information disclosed in YouTube videos created by patients with multiple sclerosis. In: Adlassnig KP, Blobel B, Mantas J, Masic I, editors. Medical Informatics in a United and Healthy Europe: Proceedings of MIE 2009, the XXII International Congress of the European Federation for Medical Informatics. Amsterdam, The Netherlands: IOS Press. 292–296. 19745316

[22] Tomes N (2006) The patient as a policy factor: a historical case study of the consumer/survivor movement in mental health. Health Affairs 25: 720–729. 16684736 10.1377/hlthaff.25.3.720

[23] Rissmiller D, Rissmiller J (2006) Open forum: evolution of the antipsychiatry movement into mental health consumerism. Psychiatric Services 57: 863–866. 16754765 10.1176/ps.2006.57.6.863

[24] Corrigan PW, Thompson V, Lambert D, Sangster Y, Noel JG, et al (2003) Perceptions of discrimination among persons with serious mental illness. Psychiatric Services 54: 1105–1110. 12883137 10.1176/appi.ps.54.8.1105

[25] Bracke P, Christiaens W, Verhaeghe M (2008) Self-esteem, self-efficacy, and the balance of peer support among persons with chronic mental health problems. Journal of Applied Social Psychology 38: 436–459.

[26] van Gestel-Timmermans H, Brouwers EP, van Assen MA, van Nieuwenhuizen C (2012) Effects of a peer-run course on recovery from serious mental illness: a randomized controlled trial. Psychiatric Services 63: 54–60. 22227760 10.1176/appi.ps.201000450

[27] Goldberg RW, Dickerson F, Lucksted A, Brown CH, Weber E, et al (2013) Living Well: an intervention to improve self-management of medical illness for individuals with serious mental illness. Psychiatric Services 64: 51–57. 23070062 10.1176/appi.ps.201200034 PMC8666111

[28] Druss BG, Zhao L, von Esenwein SA, Bona JR, Fricks L, et al (2010) The Health and Recovery Peer (HARP) Program: a peer-led intervention to improve medical self-management for persons with serious mental illness. Schizophrenia Research 118: 264–270. 20185272 10.1016/j.schres.2010.01.026 PMC2856811

[29] McKay CE, Dickerson F (2012) Peer supports for tobacco cessation for adults with serious mental illness: a review of the literature. Journal of Dual Diagnosis 8: 104–112. 22904697 10.1080/15504263.2012.670847 PMC3419592

[30] Basset T, Faulkner A, Repper J, Stamou E (2010) Lived experience leading the way: peer support in mental health. London: Together for Mental Wellbeing. Available: http://www.together-uk.org.

[31] Greene JA, Choudhry NK, Kilabuk E, Shrank WH (2011) Online social networking by patients with diabetes: a qualitative evaluation of communication with Facebook. Journal of General Internal Medicine 26: 287–292. 20945113 10.1007/s11606-010-1526-3 PMC3043192

[32] Bender JL, Jimenez-Marroquin MC, Jadad AR (2011) Seeking support on Facebook: a content analysis of breast cancer groups. Journal of Medical Internet Research 13: e16. 21371990 10.2196/jmir.1560 PMC3221337

[33] Frost JH, Massagli MP (2008) Social uses of personal health information within PatientsLikeMe, an online patient community: what can happen when patients have access to one another’s data. Journal of Medical Internet Research 10: e15. 18504244 10.2196/jmir.1053 PMC2553248

[34] Spinzy Y, Nitzan U, Becker G, Bloch Y, Fennig S (2012) Does the Internet offer social opportunities for individuals with schizophrenia? a cross-sectional pilot study. Psychiatry Research 198: 319–320. 22440545 10.1016/j.psychres.2012.02.022

[35] Veretilo P, Billick SB (2012) Psychiatric illness and Facebook: a case report. Psychiatric Quarterly 83: 385–389. 22274630 10.1007/s11126-012-9207-5

[36] Lehavot K, Ben-Zeev D, Neville RE (2012) Ethical considerations and social media: a case of suicidal postings on Facebook. Journal of Dual Diagnosis 8: 341–346.

[37] Bargh JA, McKenna KY (2004) The Internet and social life. Annual Review of Psychology 55: 573–590. 10.1146/annurev.psych.55.090902.141922 14744227

[38] Davison KP, Pennebaker JW, Dickerson SS (2000) Who talks? the social psychology of illness support groups. American Psychologist 55: 205–217. 10717968

[39] Christofides E, Muise A, Desmarais S (2009) Information disclosure and control on Facebook: are they two sides of the same coin or two different processes? CyberPsychology & Behavior 12: 341–345. 19250020 10.1089/cpb.2008.0226

[40] Ellison NB, Steinfield C, Lampe C (2007) The benefits of Facebook “friends”: social capital and college students’ use of online social network sites. Journal of Computer-Mediated Communication 12: 1143–1168.

[41] Savage J (2000) Ethnography and health care. BMJ 321: 1400–1402. 11099288 10.1136/bmj.321.7273.1400 PMC1119117

[42] Beneito-Montagut R (2011) Ethnography goes online: towards a user-centred methodology to research interpersonal communication on the Internet. Qualitative Research 11: 716–735.

[43] Gatson SN (2011) The methods, politics, and ethics of representation in online ethnography. In: Denzin N, Lincoln Y, editors. Handbook of Qualitative Research. 4 ed. Thousand Oaks, CA: Sage. 513–527.

[44] Flicker S, Haans D, Skinner H (2004) Ethical dilemmas in research on Internet communities. Qualitative Health Research 14: 124–134. 14725180 10.1177/1049732303259842

[45] McKee R (2013) Ethical issues in using social media for health and health care research. Health Policy 110: 298–301. 23477806 10.1016/j.healthpol.2013.02.006

[46] Zimmer M (2010) “But the data is already public”: on the ethics of research in Facebook. Ethics and Information Technology 12: 313–325.

[47] Charmaz K (2006) Constructing grounded theory: a practical guide through qualitative analysis. London: Sage.

[48] Corbin JM, Strauss A (1990) Grounded theory research: procedures, canons, and evaluative criteria. Qualitative Sociology 13: 3–21.

[49] Skeels MM, Grudin J (2009) When social networks cross boundaries: a case study of workplace use of Facebook and LinkedIn. Proceedings of the ACM 2009 International Conference on Supporting Group Work: 95–104.

[50] Patton MQ (1990) Qualitative evaluation and research methods. Thousand Oaks, CA: Sage.

[51] Ziebland S, Wyke S (2012) Health and illness in a connected world: how might sharing experiences on the internet affect people’s health? Milbank Quarterly 90: 219–249. 22709387 10.1111/j.1468-0009.2012.00662.x PMC3460203

[52] Zhu Q (2011) Self-disclosure in online support groups for people living with depression. Singapore: National University of Singapore. 105 p.

[53] Coursaris CK, Liu M (2009) An analysis of social support exchanges in online HIV/AIDS self-help groups. Computers in Human Behavior 25: 911–918.

[54] Mo PKH, Coulson NS (2008) Exploring the communication of social support within virtual communities: a content analysis of messages posted to an online HIV/AIDS support group. CyberPsychology & Behavior 11: 371–374. 18537512 10.1089/cpb.2007.0118

[55] Mo PKH, Coulson NS (2012) Developing a model for online support group use, empowering processes and psychosocial outcomes for individuals living with HIV/AIDS. Psychology & Health 27: 445–459. 21854088 10.1080/08870446.2011.592981

[56] Ellison NB (2007) Social network sites: definition, history, and scholarship. Journal of Computer-Mediated Communication 13: 210–230.

[57] Steffen V (1997) Life stories and shared experience. Social Science & Medicine 45: 99–111. 9203275 10.1016/s0277-9536(96)00319-x

[58] Barak A, Boniel-Nissim M, Suler J (2008) Fostering empowerment in online support groups. Computers in Human Behavior 24: 1867–1883.

[59] Davidson L, Roe D (2007) Recovery from versus recovery in serious mental illness: One strategy for lessening confusion plaguing recovery. Journal of Mental Health 16: 459–470.

[60] Entwistle VA, France EF, Wyke S, Jepson R, Hunt K, et al (2011) How information about other people’s personal experiences can help with healthcare decision-making: a qualitative study. Patient Education and Counseling 85: e291–e298. 21652162 10.1016/j.pec.2011.05.014

[61] Entwistle VA (2007) Considering ‘balance’ in information. Health Expectations 10: 307–308. 17986067 10.1111/j.1369-7625.2007.00469.x PMC5060411

